# Choline, Other Methyl-Donors and Epigenetics

**DOI:** 10.3390/nu9050445

**Published:** 2017-04-29

**Authors:** Steven H. Zeisel

**Affiliations:** UNC Nutrition Research Institute, Departments of Nutrition and Pediatrics, University of North Carolina at Chapel Hill, 500 Laureate Drive, Kannapolis, NC 28081, USA; steven_zeisel@unc.edu; Tel.: +1-704-250-5006

**Keywords:** choline, epigenetics, DNA methylation, brain development, liver cancer

## Abstract

Choline dietary intake varies such that many people do not achieve adequate intakes. Diet intake of choline can modulate methylation because, via betaine homocysteine methyltransferase (BHMT), this nutrient (and its metabolite, betaine) regulate the concentrations of *S*-adenosylhomocysteine and *S*-adenosylmethionine. Some of the epigenetic mechanisms that modify gene expression without modifying the genetic code depend on the methylation of DNA or of histones; and diet availability of choline and other methyl-group donors influences both of these methylations. Examples of methyl-donor mediated epigenetic effects include the changes in coat color and body weight in offspring when pregnant *agouti* mice are fed high choline, high methyl diets; the changes in tail kinking in offspring when pregnant *Axin(Fu)* mice are fed high choline, high methyl diets; the changes in *Cdkn3* methylation and altered brain development that occurs in offspring when pregnant *rodents* are fed low choline diets. When choline metabolism is disrupted by deleting the gene *Bhmt*, DNA methylation is affected (especially in a region of chromosome 13), expression of specific genes is suppressed, and liver cancers develop. Better understanding of how nutrients such as choline and methyl-donors influence epigenetic programs has importance for our understanding of not only developmental abnormalities but also for understanding the origins of chronic diseases.

## 1. Introduction

Our genetic code is the same in almost every cell in our body, yet all tissues do not express every gene to the same extent. For the most part, this difference between tissues occurs because there are epigenetic mechanisms that modify gene expression without modifying the genetic code [1,2,3]. This is fortunate because it permits retuning of gene expression to achieve some degree of adaptation to the environment, including the nutrition environment. The field of epigenetics is relatively new, and to date, epigenetic mechanisms involve covalent modifications to DNA (the addition of a methyl group to carbon five in cytosine [2] or the hydroxymethylation of carbon five in cytosine [4,5]); covalent modifications to histone proteins (methylation [6,7], acetylation [8], biotinylation [9] of lysine in histone tails); as well as gene expression/translation modification by microRNAs and other noncoding RNAs [10] and by nucleosome positioning/remodeling [11]. Genome-wide epigenetic status is established in early life with different cell types developing different epigenetic programs that are usually maintained throughout later life. Thus, gene expression retuning in early life can affect metabolism and organ function in later life.

## 2. Nutrient-Responsive Epigenetic Mechanisms

There are “nutrient responsive” epigenetic mechanisms that change genes’ expression, thereby modulating metabolic pathways [3,12,13,14,15,16,17,18,19,20,21,22,23]. Studies on dietary choline and other methyl-group donors provide some of the best examples of nutrient responsive epigenetic mechanisms. DNA and histone methylases are directly influenced by the availability of methyl-groups derived from diet (from choline/betaine, methyl-folate or methionine) because these are the precursors that are converted to the universal methyl-donor *S*-adenosylmethionine [24,25,26,27,28], needed for the methylation of cytosine in DNA or lysine in histones (epigenetic marks). That diet could modify DNA cytosine methylation was appreciated more than 40 years ago when it was observed that feeding rats a diet very low in choline and methionine resulted in the decreased methylation of cytosines in DNA of liver [29] which was correlated with changes in the expression of a variety of hepatic genes [30] and was linked to the spontaneous development of liver cancers on this diet [29,31,32]. Later, methylation indicator-mouse models were developed in which changes in DNA methylation during *in utero* development resulted in easily visible and stable phenotypes in mouse offspring. The mouse *agouti* gene regulates body weight, risk for diabetes and also the production of black or yellow pigment in hair. The *agouti* wildtype A mouse has a brown coat and is lean and not diabetic; the *agouti* mutant *A^vy^* has a solid yellow coat and a marked prevalence of obesity and diabetes but this phenotype can vary considerably from one mouse carrying this mutation to another, even among siblings of a single litter, because the expression of the *agouti* gene is epigenetically regulated [18]. Feeding pregnant *A^vy^/A* mice a diet high in choline, methionine, betaine, vitamin B12 and folic acid resulted in more offspring born that were brown and thin rather than possessing the normal yellow and fat phenotype for the *A^vy^/A* mouse. These phenotypes were predicted by the methylation status of *A^vy^* (methylation of cytosines in the *A^vy^* gene acts to suppress gene expression) [18,33,34]. There was a critical period in early life during which the epigenetic marks were formed (in this case, the period before birth), and after this time, restoration of a control diet did not reverse them. Mice with a different methylation indicator-gene (*Axin(Fu)*) develop kinks in their tails. When pregnant *Axin(Fu)* mice were fed diets high in choline, methionine, betaine and vitamin B12, cytosines in the gene *Axin(Fu)* were hypermethylated, gene expression was suppressed and offspring had tails with fewer kinks [16]. Again, there was a critical *in utero* time-window during which the epigenetic marks were established [16]. Other examples of choline-methyl diet modification of epigenetics include the following: insulin-like growth factor II (IGF2), which is an important growth-stimulating factor and metabolic regulator; and the expression of *IGF2*, which is negatively regulated by a long noncoding RNA made by the *H19* gene [35,36,37,38]. *H19* expression, in turn, is regulated by the methylation of a region shared by both genes called *Igf2DMR2*. When pregnant rats were fed choline-deficient diets, *Igf2DMR2* in fetal liver was hypermethylated, *H19* was inhibited and *IGF2* expression increased [39]. In people, maternal choline intake modulated epigenetic marks in the placenta; women with higher intake of choline had higher placental promoter methylation in the corticotrophin releasing hormone (*CRH*) and glucocorticoid receptor (*NR3C1*) genes and this was associated with lower placental expression of the corticotrophin-releasing hormone [40]. Though much of the epigenome is established during early life, some epigenetic marks are modifiable in later life. For example, methylation levels of specific CpG sites from *Srebf2*, *Agpat3*, *Esr1* and *Fasn* promoter regions in liver were changed when rats were fed a high choline (high methyl) diet, and these effects were gender dependent [41,42]. Why some epigenetic marks are fixed in early life and others are plastic is not understood.

## 3. Choline, Epigenetics and Fetal Development

The dietary choline-mediated effects on epigenetic marks are not limited to the genes discussed above; feeding pregnant mice a diet low in choline decreased cytosine methylation in the gene cyclin dependent kinase 3 (*Cdkn3*), resulting in increased expression of this gene that inhibits cell cycling [43]. These diet-mediated epigenetic changes in *Cdkn3* were associated with decreased proliferation of neural progenitor cells in the fetal brain [43,44]. This is especially interesting because maternal dietary choline is essential for normal brain development.

There is extra demand for choline during pregnancy and this increases the choline requirements of the mother and often exceeds the capacity for endogenous production of choline (in the form of phosphatidylcholine) in the liver [45]. Estrogen can induce the gene *PEMT* encoding the enzyme catalyzing the production of phosphatidylcholine in liver, and this means that young women are somewhat less dependent on dietary intake of choline than are men [46,47]. Unfortunately, almost half of women have a gene variant that blocks estrogen-induction of the capacity to make their own choline [46,47]; these women may be especially sensitive to dietary choline variations during pregnancy. The placenta and mammary gland deliver large amounts of choline from mother to the fetus and infant, respectively [48,49]. This results in plasma and tissue choline concentrations that are much higher in the fetus and young infant than they are in adults [50,51]. 

Maternal supply of choline to the fetus and infant is important for brain development. In rodents, decreased supply of choline during a critical window of brain development (in the rodent, days 11–17 of gestation) results in decreased neurogenesis in the brain cortex [52] and hippocampus [44,53]. Lower maternal choline intake results in smaller fetal brains, with markedly diminished numbers of fetal progenitor cells in the germinal layers of both the cortex and hippocampus [52]. Also, with lower maternal choline intake, there is a loss of the normal layering of the developing fetal cortex because neural progenitor cells differentiate too early, forming layer 6 of the cortex, and leave too few remaining progenitors to form the five other later-born layers of the brain [52]. These six different layers connect to different neural pathways in the brain, and when they are not formed properly, these pathways are disrupted. One mechanism for this effect of choline is mediated by changes in the epidermal growth factor (EGF) signaling (required for normal timing of differentiation) in the developing cortex. Low choline availability to the fetal brain results in decreased production of the receptor for EGF [52]. Similarly, low maternal choline intake results in premature differentiation of neural progenitors in the fetal hippocampus [54,55,56,57]. This area of the brain is important for memory function. If pregnant rats are fed a diet supplemented with choline, their offspring perform better in tests of visuospatial and auditory memory [58,59,60] and this improvement lasts for their lifetime. Conversely, when pregnant rats are fed diets low in choline, their offspring have worse visuospatial and auditory memory [61]. Babies born from women who eat more choline during pregnancy perform better at 7 years of age in visuospatial memory testing [62], suggesting that the effects of choline in rodents may also apply to people. However, one observational study [63] reported that there was no association between cord blood choline concentrations and scores on standard intelligence tests in 5 year olds.

In addition to influencing brain neurogenesis, fetuses from pregnant mice fed a low choline diet have fewer small blood vessels in their hippocampi when compared to fetuses from mothers fed a normal or high choline diet [64]. Endothelial progenitor cells in brains of the low choline group, responding to increased expression of *Vegfc* and *Angpt2*, differentiate prematurely and stop dividing, with resulting reductions in blood vessel formation [64]. The increased expression of *Vegfc* and *Angpt2* is associated with the decreased methylation of cytosines within these two genes [64].

Thus, choline has an important role in regulating the development of the brain, and epigenetic mechanisms that likely contribute to the underlying pathways for these effects.

## 4. Choline, Epigenetics and Liver Cancer

In addition to its role in brain development, choline and other methyl-donors also have a role in carcinogenesis. Rodents fed low choline, low methyl diets develop liver cancers [65,66,67,68]. A meta-analysis of 11 studies in people calculated that diets low in choline increased the overall relative risk for developing cancer [69] with the largest reported effects found for lung (30% increase; also see [70]), nasopharyngeal (58% increase; also see [70]) and breast cancer (60% increase; also see [71]). An increment in diet intake of 100 mg/day of choline and betaine (a metabolite derived from choline) helped reduce cancer incidence by 11% [69].

It is possible that these effects of choline are mediated by epigenetic mechanisms [72]. Choline-methyl-deficient diets result in decreased hepatic concentrations of *S*-adenosylmethionine, and increased concentrations of *S*-adenosylhomocysteine in the livers of male rats and mice [73]. Low *S*-adenosylmethionine combined with high *S*-adenosylhomocysteine concentrations act to inhibit methyltransferases’ activity [74]. *S*-adenosylhomocysteine has high affinity for DNA methyltransferases (DNMTs) and competes for binding of *S*-adenosylmethionine, thereby inhibiting DNMT enzymatic activity [75]. Choline-methyl-deficient diets result in decreased DNA methylation [72,30] at many sites including the oncogene c-*myc* [76], but also in increased methylation at other cytosines (including the hypermethylation of several tumor-suppressor genes such as *p53*, *p16^INK4a^*, *PtprO*, *Cdh1*, and *Cx26*) [72,77,78,79]. Histone methylation is also affected by methyl-deficient diets. Feeding a choline-methyl-deficient diet results in decreased hepatic histone H3 lysine 9 trimethylation (H3K9me3) and histone H4 lysine 20 trimethylation (H4K20me3) [72]. In addition, several histone methyltransferases are down-regulated (Suv39h1, Prdm2/Riz1, and Suv420h2) [72]. Finally, down-regulation of microRNAs (miR-122 and miR-29), and up-regulation of miR-34a, miR-155, and miR-221 occur in methyl-deficient liver [72]. These microRNAs act to modify the expression and translation of specific gene products.

When a key gene (*Bhmt*; betaine homocysteine methyltransferase) in choline metabolism is deleted in mice, these mice develop hepatocarcinomas [80]. The betaine-dependent methylation of homocysteine (by BHMT) is important for maintaining concentrations of *S*-adenosylmethionine, and for removing *S*-adenosylhomocysteine in liver and for maintaining normal patterns of DNA methylation [80]. Deleting *Bhmt* causes mostly loss of methylation at 63 differentially methylated cytosines. Of these, 33 (mostly hypomethylated) are located in one locus spanning 15.5 Mb on chromosome 13 (from 93.5 to 109 Mb (mm9), which includes the *Bhmt* gene located at chr13: 94,386,846–94,407,713) [80]. It is possible that the sensitivity to DNA methylation perturbations that we observe in chromosome 13 is a regulatory mechanism meant to sense methyl-metabolism, as not only *Bhmt* but other 1-carbon metabolism genes are located there: dimethylglycine dehydrogenase (*Dmgdh*) at chr.13: 94,444,391, methionine synthase reductase (*Mtrr*) at chr.13: 68,699,657 and 5-methyltetrahydrofolate-homocysteine methyltransferase (*Mtr*; also in chromosome 13 but outside the locus at chr.13: 12,279,086) [80]. The altered DNA methylome in *Bhmt*-null mice is associated with changes in gene expression in liver; of 18 differentially expressed genes, five are located on chromosome 13, with four out of the five falling within the chromosome 13 hypomethylated block: *Arsb, F2rl2, Iqgap2*, and *Enc1* [80]. *Iqgap2* belongs to a family of scaffolding proteins, which mediate Rho GTPase and Ca^2+^/calmodulin signaling in regulating multiple cellular processes, such as cell adhesion, motility, and exocytosis [81]; *Iqgap2*-null mice develop spontaneous hepatocellular carcinomas [82]. *F2rl2* is a member of the protease-activated receptor-3 (PAR-3) family and is involved in homeostasis, adhesion, proliferation, and migration [83,84]; *F2rl2* binds to signal transduction proteins and transcription factors involved in carcinogenesis [85,86].

Thus, choline and methyl metabolism has an important role in regulating carcinogenesis, and epigenetic mechanisms likely contribute to the underlying pathways for these effects.

## 5. Choline Intake Is Marginal in Many People

Choline is a required nutrient and in 1998, an Adequate Intake (AI) and a Tolerable Upper Limit (UL) for choline was established [87]. In 2016, the US Food and Drug Administration (FDA) set a Recommended Daily Intake (RDI) for choline based on the AIs as part of the new Nutrition Facts label for packaged foods (published in the Federal Register on May 27, 2016; FDA-2012-N-1210-0875, Federal Register Number:2016-11867). The US AI/RDI varies by age and gender, but is 550 mg/day in adult men and 425 mg/day in adult women (450 mg/day in pregnant and 550 mg/day lactating women). The European Food Safety Authority recently established slightly different recommendations for Adequate Intake levels for choline (European Food Safety Authority, Parma, Italy, 17 August 2016).

The US Department of Agriculture maintains a database of the choline content of foods (https://www.ars.usda.gov/northeast-area/beltsville-md/beltsville-human-nutrition-research-center/nutrient-data-laboratory/docs/usda-database-for-the-choline-content-of-common-foods-release-2-2008/); foods such as eggs and meats contain more choline than do most plant sources. There is a wide variation in choline intake in the diet; the US National Health and Nutrition Examination Survey (NHANES 2009–2012) reports that only 11% of adult Americans achieve the Adequate Intake level of choline, with mean intake being 300 mg/day (10%ile being 200 mg/day; 90%ile being 500 mg/day) [88]. Similar ranges of intake were reported in the Framingham Offspring Study [89], the Atherosclerosis Risk in Communities study [90,91] and the Nurse’s Health Study [92]. Intake of choline is even lower in low-income countries such as Jamaica and The Gambia [93,94].

## 6. Conclusions

Choline is an essential nutrient with functional relevance in a wide array of biological pathways including epigenetic modulation of gene expression. Timing of nutrient availability is important in determining epigenetic outcomes; changes in availability during early life potentially having a greater effect than changes occurring later in life. The Developmental Origins of Health and Disease (DOHaD) hypothesis [95] is that diet in early life influences developmental plasticity and alters susceptibility to adult cardiovascular disease, type 2 diabetes, obesity [96], neuropsychiatric diseases [97], immune/inflammatory diseases [98] and cancer [99]. Epigenetic mechanisms are likely a major mechanism of DOHaD [100]. Thus, better understanding of how nutrients such as choline and methyl-donors influence epigenetic programs has importance for our understanding of not only developmental abnormalities but also for understanding the origins of chronic diseases.
